# Persistent pain associated with socioeconomic and personal factors in a Sami and Non-Sami population in Norway: an analysis of SAMINOR 2 survey data

**DOI:** 10.1080/22423982.2020.1787022

**Published:** 2020-08-11

**Authors:** Elin Damsgård, Gyrd Thrane, Nils Fleten, Johan Bagge, Tore Sørlie, Audny Anke, Ann-Ragnhild Broderstad

**Affiliations:** a Department of Health and Care Sciences, UiT the Arctic University of Norway, Tromsø, Norway; b Department of Community Medicine, UiT the Arctic University of Norway, Tromsø, Norway; c UiT the Arctic University of Norway, Tromsø, Norway

**Keywords:** Persistent pain, ethnicity, Sami, indigenous, Norway

## Abstract

In international studies, higher prevalence of persistent pain has been reported in indigenous populations compared to majority populations. The present study aimed to determine the prevalence of persistent pain within a Sami and a non-Sami population in northern Norway, with adjustment for the confounding factors of age, sex, marital status, education, income, mental health, smoking status and ethnic background. Using SAMINOR 2 survey data including Sami and non-Sami populations, we analysed 5,546 responses, from individuals aged 40–79 years, to questions concerning persistent pain (≥ 3 months). In total, 2,426 (43.7%) participants reported persistent pain with differences between Sami women and non-Sami women (44.1% versus 51.1%, respectively), but none between Sami men and non-Sami men (38.7% versus 38.2%, respectively). Elderly Sami women were less likely to report persistent pain than were elderly non-Sami women. In men, no ethnic differences in pain were observed according to age-group. Marital status, education levels, household income, psychological distress, and smoking status did not influence the association between ethnicity and pain. Pain severity and location did not differ between Sami and non-Sami participants. In this study, we found only minor ethnic differences in persistent pain. Similar living conditions and cultural features may explain these findings.

## Background

Persistent pain has been shown to have a significant effect on health and function and has been reported to be the most common reason given for sick leaves and disability pensions in Norway [1]. Moreover, population-based studies have shown increasing prevalence rates for persistent pain [1,2].

The experience and expressions of persistent pain may be influenced by biological, cultural, psychological, and social factors [3]. Low income and low levels of education have been associated with higher prevalence rates for persistent pain [4]. Personal risk factors include female sex, higher age, being unmarried, or living alone [5], and the prevalence of pain is higher in smokers than in non-smokers [6]. Population-based studies have reported that psychological factors such as anxiety and depression, as well as resilience, are closely associated with persistent pain [7,8].

International studies have suggested that persistent pain is a greater issue among indigenous peoples than among the general population [9–11]; however, the results are inconclusive [12]. Differences in health status have been reported to be related to inequities in social and economic conditions; however, international studies have also found differences between ethnic groups in terms of reporting, perception, and management of pain [9,13]. Therefore, assuming an association between expression and experiences of pain, it is relevant to consider which cultural factors characterise different ethnic groups.

Northern Norway is a multicultural society with the largest proportion of Sami people among those living in Norway. The Sami people are recognised as Norway’s indigenous people and the original Sami landscape, *Sapmi*, covers the northern regions of Norway, Sweden, and Finland [14]. Due to incomplete registrations, migration, and social processes, the number of people with a Sami background is difficult to determine. A previous policy of Norwegianization [15] was aimed at making the Sami people relinquish their language and identity. Consequently, some people no longer wanted to appear in public as Sami, as they considered it to be shameful, and Minde [15] referred to this experience as “cultural pain.” Regarding the expression of illness, Sami traditions and culture have several features that can affect the experience and communication of pain. For example, health issues, and mental health issues in particular, are topics you “don’t talk about” [16].

For people of Sami background, several factors reported as being strongly associated with persistent musculoskeletal pain in international studies are represented. For example, members of the Sami population have experienced discrimination, and perceived discrimination has been shown to be associated with poor mental health [17] and back pain [18]. In one Norwegian study, participants of Sami ethnicity who reported perceived discrimination more frequently reported musculoskeletal pain compared to the general population [19]. Further, individuals of Sami background have reported poorer self-perceived health, partly associated with lower socioeconomic status [20]. However, regarding socioeconomic conditions, higher education levels are more common among women between 35 and 50 years old with a Sami background than among non-Sami women from the same area [21]. Previous Norwegian studies that included pain variables found that childhood violence was associated with adult pain regardless of ethnicity [22], and in a study of Sami and non-Sami adolescents, Eckhoff and Kvernmo found no ethnicity-related differences in musculoskeletal pain [23]. Among reindeer herders in Sweden, the prevalence rates for musculoskeletal pain have increased over recent decades, which has been considered a consequence of psychosocial, physical, and socioeconomic risk factors; however, there are only about 2000 Sami reindeer husbandries in each of northern Norway and Sweden [24].

No previous studies have investigated the prevalence of persistent pain in Sami and non-Sami people in a Norwegian multiethnic population or examined the association between persistent pain and socioeconomic and personal factors among different ethnic groups. The present study aimed to determine the prevalence of persistent pain within a Sami and non-Sami population in northern Norway, with adjustment for the confounding factors of age, sex, marital status, education, income, mental health, smoking status and ethnic background.

## Methods

We used data from the population-based survey on Health and Living Conditions in Regions with Sami and Norwegian Populations, (the SAMINOR survey), as organised by the Centre for Sami Health Research at the University of Tromsø – The Arctic University of Norway. Three SAMINOR surveys have been conducted to date. The SAMINOR 1 survey was conducted between 2003 and 2004. A second survey followed in 2012–2014 comprising two separate surveys: the SAMINOR 2 Questionnaire Survey (2012), and the SAMINOR 2 Clinical Survey (2012–2014). This paper is based on data from the SAMINOR 2 Clinical Survey. Details on the data collection process are published elsewhere [25].

SAMINOR 2 was undertaken in ten municipalities that had all been included in SAMINOR 1. All inhabitants aged 40–79 years registered in the National Registry as residents in one of the ten municipalities were invited to participate in the clinical survey. The regional Committee for Medical and Health Research Ethics of North Norway approved the survey and all participants provided written informed consent. Of 12,455 inhabitants, 6,004 (48.2%) participants in SAMINOR 2 attended a clinical examination, provided informed consent to participate in medical research, and agreed to have their data linked to other surveys and registers. Data were collected through self-administered questionnaires, clinical examinations, and blood sampling.

### Measures

#### Ethnicity

In total, the participants were asked 11 questions concerning their ethnicity, including: “*What language(s) do/did you, your parents, and your grandparents use at home?”* and “*What is your, your father’s, and your mother’s ethnic background?”* as well as a question concerning self-perceived ethnicity: “*What do you consider yourself to be?”* For all questions, the response options were Norwegian, Sami, Kven (of Finnish origin), or Other. The questions were answered separately for each relative, and multiple choices were allowed. Respondents were categorised as Sami if they responded that they considered themselves Sami, even if they considered themselves multiethnic. All other respondents were categorised as non-Sami [25].

#### Persistent pain

Persistent pain was defined as pain lasting for at least 3 months. Respondents were required to answer whether they currently experienced pain that had lasted for ≥ 3 months (yes/no). This yes/no questionnaire is easy to answer and has been used in other population-based studies on pain in Norway [26].

#### Pain severity

Respondents who reported pain for the last 3 months or more were asked to mark their pain severity during the last week, using a numeric rating scale (NRS) ranging from 0 (*no pain*) to 10 (*the worst pain imaginable*). The NRS has been shown to be applicable for the assessment of pain severity in most settings [27]. Several studies, including studies of the general population, have investigated categorisations and cut-off points for mild, moderate, and severe pain [28,29]. Based on results from those studies, we categorised pain severity as follows: no pain (NRS = 0), mild pain (NRS = 1–4), moderate pain (NRS = 5–7), and severe pain (NRS = 8–10).

#### Pain location

The question on pain location offered three possible answers: “the neck,” “the lower back,” or “other.” Respondents were asked to mark one region, by ticking the option, in which their pain was most severe.

#### Psychological distress

A shortened 5-item version of the Hopkins Symptoms Check List (HSCL-5) was used to measure global psychological distress, primarily anxiety and depression (25). The HSCL-5 measured respondents’ feelings during the last 4 weeks in terms of nervousness, anxiousness, hopelessness, apprehension, and feelings of dejection. Each item was rated on a 4-point scale ranging from “*not at all*” (1 point) to “*extremely*” (4 points). The HSCL-5 score was calculated by summing the items and dividing the total score by 5. A cut-off point of 2 has previously been recommended as a valid predictor of psychological distress [30]. The questionnaire has previously been validated, with similar cut-off points for both Sami and non-Sami populations [31] with response sheets indicating ≥2 missing items excluded. Missing values were replaced with the mean value of the remaining items [30].

#### Socioeconomic status

Educational levels were categorised as low (<10 years), medium (10–13 years), and high (>13 years). In terms of household incomes (2013/2014), participant incomes were categorised in Norwegian Crowns (NOK) in terms of yearly income as either low (NOK <150 000), medium-low (NOK 150 000–450 000), medium-high (NOK 451 000–600 000), or high (NOK >600 000). Marital status was categorised as single or married/living with someone else.

#### Smoking status

Respondents were asked to answer “Yes” or “No” to whether they smoked on a daily basis, or whether they had been previous smokers.

#### Final sample

**Figure 1.**
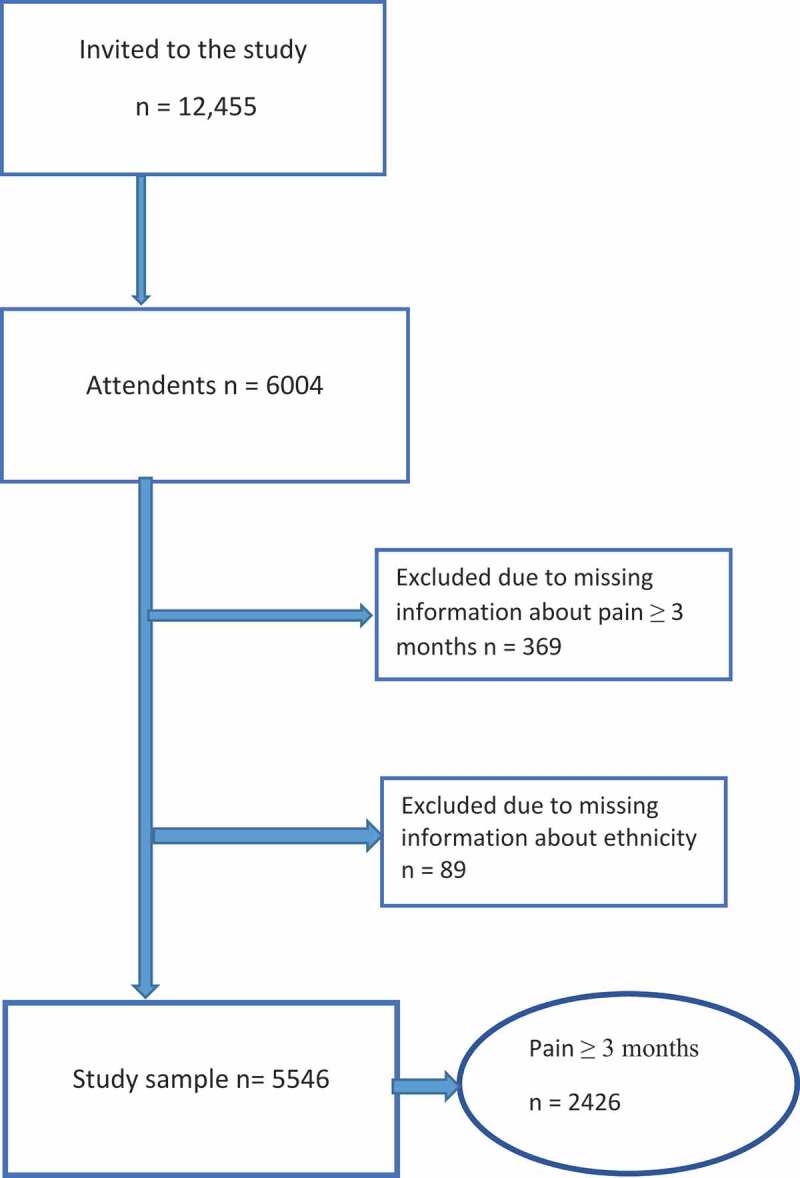
Flowchart of participant selection.

Respondents with missing information concerning ethnicity (*n* = 89) and persistent pain (*n* = 369) were excluded, leaving 5,546 (44.5% of invitation sample) respondents eligible for analysis in our study (Figure 1). Missing values existed for *marital status* (*n* = 52), *education* (*n* = 210), *household income* (*n* = 1,289), *psychological distress* (*n* = 1,224), *pain severity* (*n* = 121), and *pain location* (*n* = 683).

### Statistical analyses

The R language for statistical computing version 3.6.1 was used for data analyses [32]. Frequencies and descriptive statistics were calculated to describe the study population with regard to socio-demographic variables, persistent pain, smoking status, and mental health. As health variables are known to differ between the sexes, we analysed men and women separately. We analysed the full study sample using Chi-square tests and logistic regression analyses to assess and compare the proportions of respondents reporting persistent pain in the non-Sami and Sami populations. We first analysed how ethnicity influenced the odds ratio for persistent pain. With ethnicity as the independent variable, we assessed the confounding effect of age, marital status, education, income, psychological distress, and smoking status in separate tri-variate models. A −2log likelihood test was used to determine statistical interference. We then assessed the interactions between ethnicity and each co-variable by including an interaction term in each model. We assessed pain severity and pain location in the smaller group of respondents who had confirmed the presence of persistent pain and who had responded to the questions concerning pain severity and location. We investigated these data using frequencies and chi-square analyses and we compared data according to ethnicity and sex. The level of significance was set at .05.

## Results

A total of 5,546 of 6,004 participants answered questions concerning pain and were included in the study sample (Figure 1). There were significantly more men (93.8%) than women (91.1%) who responded to the pain questions (*p* < .001). Respondents to the pain questions were significantly younger (−5.6 years, *p* < .001), had higher education levels (2.2 years, *p* < .001), and higher income ($\chi^{2}$ = 56.1, df = 6, *p* < .001) compared with non-respondents.

In the study sample, 2,119 (38.2%) identified themselves as Sami. As shown in Table 1, more Sami were single (*p* < .001) and had a lower income compared to non-Sami women and men (*p* < .001). Regarding education, more Sami women were found to have a higher level of education compared to non-Sami women (*p* < .001), whereas Sami men were found to have lower education levels than did non-Sami men (*p* = .004). There were no ethnic differences in smoking habits. Sami respondents reported higher levels of psychological distress compared to non-Sami respondents (Table 1).

**Table 1.** Descriptive data in Sami and non-Sami women and men in the study sample, including p-values for ethnic differences.

	Women			Men		
	Sami	Non-Sami	p-value	Sami	Non-Sami	p-value
N	1139	1829		980	1598	
Age, years n(%)						
40–49	284 (24.9)	442 (24.2)	0.16	200 (20.4)	317 (19.8)	0.347
50–59	341 (29.9)	499 (27.3)		262 (26.7)	392 (24.5)	
60–79	514 (45.1)	888 (48.6)		518 (52.9)	889 (55.6)	
Marital status n (%)						
Cohabitant	739 (64.9)	1331 (72.8)	<0.001	687 (70.1)	1225 (76.7)	<0.001
Single	391 (34.3)	482 (26.4)		281 (28.7)	358 (22.4)	
Household income n(%)						
Low (<300 t)	140 (15.8)	170 (12.4)	0.002	131 (17.0)	150 (12.3)	0.003
Medium low (300 t – 450 t)	189 (21.3)	255 (18.5)		132 (17.1)	179 (14.7)	
Medium high (450 t-600 t)	208 (23.4)	300 (21.8)		158 (20.5)	252 (20.6)	
High (>600 t)	351 (39.5)	651 (47.3)		351 (45.5)	640 (52.4)	
Education, years n(%)						
<10 years	287 (26.5)	445 (25.2)	<0.001	327 (34.9)	446 (28.8)	0.004
10–12 years	252 (23.3)	540 (30.6)		276 (29.4)	526 (33.9)	
13 or more years	542 (50.1)	782 (44.3)		335 (35.7)	578 (37.3)	
Smoking n(%)						
Yes	689 (60.5)	1097 (60.0)	0.822	653 (66.6)	1068 (66.8)	0.964
No	435 (38.2)	707 (38.7)		321 (32.8)	521 (32.6)	
Psychological Distress n(%) (HSCL-%) n(%)						
1.0-1.9	761 (66.8)	1265 (69.2)	0.008	668 (68.2)	1111 (69.5)	0.002
>2.0	131 (11.5)	154 (8.4)		90 (9.2)	92 (5.8)	

(Chi- square analyses)

In the study sample, 2,426 (43.7%) respondents reported having current pain which had lasted for ≥3 months (Figure 1). Of these, 2,305 (95.0%) answered the question about pain severity, and 1,743 (71.8%) indicated the pain location. In the Sami population, a smaller proportion, (*n* = 881, 41.6%) reported persistent pain compared to the proportion of non-Sami respondents (*n* = 1545, 45.1%, $\chi^{2}$ = 6.54, df = 1, *p* = .011).

As shown in Table 2, “mild pain” was most commonly reported among the non-Sami and Sami respondents, and there were no differences between Sami and non-Sami respondents concerning pain severity. Regarding pain location, “other” was most commonly reported, and there were no differences between Sami and non-Sami respondents (Table 2). However, we found sex differences. In the Sami population, women more frequently reported “moderate” and “severe” pain compared to men (*p* = .036). Women reported significantly more neck pain, and men more low back pain in both the Sami (*p* = .027) and the non-Sami populations (*p* = .004).

**Table 2.** Pain severity (Numeric Rating Scale) and pain location in respondents with persistent pain, stratified by ethnicity and sex. P-values for ethnic differences are based on Chi – Square analyses. The table shows the total number of respondents with pain, and the proportion of respondents who reported pain severity and pain location.

	Women			Men		
	Sami	Non-Sami	p	Sami	Non-Sami	p
Respondents with pain	502	934		379	611	
Pain Severity last week, n (%)			0.322			0.083
No Pain (NRS = 0)	6 (1.3)	4 (0.5)		2 (0.6)	6 (1.0)	
Mild pain (NRS 1–4)	224 (47.4)	403 (45.6)		197 (54.7)	272 (46.3)	
Moderate pain (NRS 5–7)	192 (40.6)	382 (43.2)		139 (38.6)	268 (45.6)	
Severe pain (NRS 8–10)	51 (10.8)	95 (10.7)		22 (6.1)	42 (7.1)	
Missing	29	50		19	23	
Pain location, n (%)			0.075			0.584
Neck	71 (18.9)	131 (20.1)		38 (14.1)	60 (13.5)	
Lower back	71 (18.9)	159 (24.3)		73 (27.1)	137 (30.8)	
Other	234 (62.2)	363 (55.6)		158 (58.7)	248 (55.7)	
Missing	126	281		110	166	

We found that age was significantly related to pain in men, as older men reported less pain than did younger men ($\chi^{2}$ = 18.1, df – 2, *p* < .001). Regarding marital status, there was no difference between the proportion of married and single women reporting pain, in either Sami or non-Sami respondents. Men who lived alone reported pain more frequently than men who lived with a partner ($\chi^{2}$= 3.99, df = 1, *p* = .046), and marital status contributed to the reporting of pain (p = .042). The level of education was significantly related to pain in women ($\chi^{2}$ = 6.67, df = 2, *p* = .036) but not in men ($\chi^{2}$ = 4.75, df = 2, *p* = .093). Household income was associated with pain in in both women ($\chi^{2}$ = 41.3, df = 1, *p* = .002) and men ($\chi^{2}$ = 9.41, df = 3, *p* = .024). As anticipated, psychological distress was significantly associated with pain, in both women ($\chi^{2}$= 41.3, df = 1, *p* < .001) and men ($\chi^{2}$ = 37.28, df = 1, *p* < .001). Smoking was not associated with pain.

Table 3 shows the proportion of respondents reporting persistent pain, according to age groups and other covariates, stratified in terms of sex and ethnicity. In women, we found a significant interaction between ethnicity and age ($\chi^{2}$ = 10.1, df = 4, *p* = .039), as elderly Sami women were less likely to report pain than were elderly non-Sami women; no differences were observed in men. As shown, neither marital status nor educational level influenced the relationship between ethnicity and persistent pain in either sex. Pain reporting in the various income groups showed a somewhat different pattern between Sami and non-Sami respondents. In Sami women, the proportion of those with pain decreased less between low-income and high-income groups (from 48.6% to 43.6%, respectively,$\chi^{2}$ = 1.77, df = 3, *p* = .622) compared to non-Sami women (from 62.4% to 48.2%, $\chi^{2}$ = 11.89, df = 3, *p* = .007). Household income did not influence the relationship between ethnicity and pain in women. In contrast to our findings concerning women, more Sami men than non-Sami men in both low-and high-income groups reported persistent pain, and the proportion of both Sami and non-Sami men with pain decreased significantly between low- and high-income groups ($\chi^{2}$ = 10.68, df =3, *p* = .014 and $\chi^{2}$ = 10.17, df = 3, *p* = .017, respectively). When we included household income in the regression model, there was an increased odds ratio for pain in Sami men, but this result was not significant.

**Table 3.** The table shows the proportion of respondents who reported pain, in age groups, marital status, educational level, household income, psychological distress and smoking status, stratified by sex and ethnicity, and the adjusted OR for persistent pain in Sami for each of the covariates. *Unadjusted OR.

	Women (n, %)						Men (n, %)					
	Sami		Non-Sami		Adj OR	p-value	Sami		Non-Sami		Adj OR	p-value
Respondents with pain	502	44.1%	934	51.1%	0.76*	<0.001	379	38.7%	611	38.2%	1.02*	0.824
Age												
40–49	137	48.2%	216	48.9%	0.75	<0.001	92	46.0%	119	37.5%	1.01	0.920
50–59	162	47.5%	267	53.5%			110	42.0%	179	45.7%		
60–79	203	39.5%	451	50.8%			177	34.2%	313	35.2%		
Marital status												
Married/Cohabitant	326	44.1%	683	51.3%	0.74	0.002	261	38.0%	451	36.8%	1.01	0.950
Single	173	44.2%	245	50.8%			113	40.2%	154	43.0%		
Missing	3	33.3%	6	37.5%			5	41.7%	6	40.0%		
Education												
Low (< 10 years)	124	43.2%	232	52.1%	0.77	<0.001	130	39.8%	184	41.3%	1.01	0.945
Medium (10–12 years)	120	47.6%	295	54.6%			108	39.1%	208	39.5%		
High (13 or more)	236	43.5%	381	48.7%			124	37.0%	202	34.9%		
Missing	22	37.5%	26	41.9%			17	40.5%	17	35.4%		
Gross income												
Low (≥300 t)	68	48.6%	106	62.4%	0.73	0.001	71	54.2%	64	42.7%	1.08	0.410
Medium low (301 t – 450 t)	90	47.6%	138	54.1%			52	39.4%	85	47.5%		
Medium high (451 t-600 t)	90	43.3%	162	54.0%			67	42.4%	107	42.5%		
High (>600 t)	153	43.6%	314	48.2%			135	38.5%	227	35.5%		
Missing	101	40.2%	214	47.2%			54	26.0%	128	34.0%		
HSCL-5												
1.0–1.8	322	42.3%	631	44.9%	0.73	<0.001	262	39.2%	418	37.6%	1.04	0.682
2.0–4.0	82	62.6%	110	71.4%			53	58.9%	59	64.1%		
Missing	98	39.7%	193	47.1%			64	28.8%	134	33.9%		
Smoking												
Yes	316	45.9%	576	52.5%	0.75	<0.001	258	39.5%	421	39.4%	1.02	0.836
No	179	41.1%	347	49.1%			118	36.8%	186	35.7%		
Missing	7	46.7%	11	44.0%			3	50.0%	4	44.4%		

There were more non-Sami respondents with pain in the high stress group (HCSL score > 2) compared to Sami respondents of both sexes, and the proportion of current smoking non-Sami women with pain was higher compared to Sami women. However, neither psychological distress nor being a current smoker influenced the relationship between ethnicity and pain (Table 3).

## Discussion

This study investigated the prevalence of persistent pain within a Sami and non-Sami population in northern Norway, with adjustment for the confounding factors of age, sex, marital status, education, income, mental health, smoking status, and ethnic background. We found that Sami women were less likely to report persistent pain compared to non-Sami women in the same age group. Marital status, level of education, household income, psychological distress, and smoking status did not influence the relationship between ethnicity and persistent pain.

The reported prevalence of current persistent pain (43.7%) in our study was high compared to previous international studies, which have reported prevalence rates for pain ranging from 12% to 32% [33,34]; however, our results were in line with findings from epidemiological research in Norway that showed an overall prevalence rate concerning musculoskeletal complaints of 47.9% [2]. An even higher prevalence was reported in a Tromsø study, with 62% of the participants reporting pain, of whom 45.6% reported mild pain and 16.4% reported severe pain [35]. Our study did not distinguish between different pain origins, and consequently the result of persistent pain cannot be examined by origin; however, it may be likely that neck and lower back pain is of musculoskeletal origin. Differences in populations, pain measurements, and non-consistency in definitions of persistent pain are known problems in epidemiological pain research [36,37] that make comparisons challenging.

Approximately 40% of the respondents described their pain severity as moderate (NRS 5–7), which appears to be high for a population-based study. There are also issues with assessment of pain severity, as the diversity in assessment tools is significant [38]. However, our results were in accordance with those of Rustøen et al. [39], who found an average NRS score of 5.2 in a Norwegian population with pain, and those of Schopflocher et al. [38], who found that 18.9% of Canadian respondents scored > 5 for pain severity. Regarding pain location, the majority of respondents reported “other.” The higher age in this population may have resulted in more respondents reporting pain from various parts of the body, for example, headache and chest pain. In line with Rustøen [39], both Sami and non-Sami women reported more neck pain compared to men. Sex differences regarding lower back pain appear inconsistent [40], and the higher prevalence rate among men in our study may be associated with workplace exposure, as this study took place in rural areas where work is more likely to be physically demanding.

We found that a smaller proportion of elderly respondents reported persistent pain than did younger respondents. Musculoskeletal pain has been shown to increase with age, with a tendency to diminish in the oldest populations [41]. Other studies have also found that higher age did not predict pain in the elderly [26,42]. The proportion of elderly women with pain (Sami 39.5% and non-Sami 50.8%) is remarkable compared to the findings of a Swedish study, in which 64.5% of women aged > 60 years reported pain [43]. It is also notable when compared to the Tromsø study, in which 49.8% of women aged 70 −79 years reported mild pain, and 23.5% of women in the same age group reported severe pain [35]. It may be that elderly respondents in our study have grown up in societies with limited access to medical aid, and may thus be used to living with pain [44]. This could have affected their responses to the pain question. Further, physical activity seems to reduce pain in elderly populations [45]. In a study of physical activity in northern Norway, Sami men and women aged 40–62 years reported more general physical activity than non-Sami individuals [46]. Both groups reported activities related to farming, fishing, walking (berry picking and reindeer herding), and the Sami participants, in particular, did not distinguish between work and leisure time activities [46]. This traditional life-style may have contributed to less pain in the elderly. More updated research in this area would be useful in future studies.

Marital status was associated with persistent pain in our study, as men who lived alone reported more pain than men who lived with a partner. Being married is a protective factor for pain and mental stress in both sexes when the marriage is non-conflicted [47,48]. We found no other studies with findings that are similar to our results. Marriage, as well as pain, is associated with social support [48], and men in this study who were living alone may have experienced less social support.

Regarding education and household income, we found that the level of education was associated with pain in women, but not in men. Persistent pain has been shown to be associated most consistently with education levels in women, and with occupational class in men [49]. In our study, there was a large proportion of both Sami (50.1%) and non-Sami (44.3%) women with higher education. This could have contributed to the minor difference in persistent pain between Sami and non-Sami women. However, the difference in reported pain was found in elderly women, and other explanations are more likely for that population. Household income was associated with pain in both women and men, which is in line with previous research [35]. We observed a different gradient in Sami women compared to non-Sami women, as the proportion of respondents with pain decreased less from the low- to the high-income group in Sami women compared to non-Sami women. It thus seems like Sami women were less vulnerable to pain when examined in the context of income. This interesting trend should be examined in a larger cohort in the future.

In the binomial logistic regression analyses, the only significant difference we found between Sami and non-Sami responders was that elderly Sami women were less likely to report persistent pain compared to non-Sami women. This was an unexpected finding, particularly in the context of studies in which elderly Sami women reported poorer health conditions compared to the general population, and self-reported health is associated with pain [K. L. 20]. Hence, the analyses showed only minor ethnic differences in reports of persistent pain, despite the fact that the Sami respondents were more frequently single, had lower education (men), lower income, and higher levels of psychological distress.

The interpretation of these interesting findings is quite challenging. One possible explanation is that our results reflect cultural features as protective factors. Pain is a complex experience that is characterised as the interrelationship between biological, psychological, and sociocultural aspects, and the experience and expression of pain may be culturally influenced. In a study of social support among older American indigenous peoples, Conte et al. found that less persistent pain was linked to higher social support, and that increased age, being married, and being female were factors associated with high social support [50]. In the Sami culture, closeness to relatives and the presence of an extended family is important, especially among elderly Sami women [51]. These features may promote resilience and mitigate the effect of symptoms such as pain. We found that psychological distress was associated with persistent pain in both women and men; interestingly, however, there were more non-Sami than Sami responders with pain in the high stress group. Friborg et al. [52] found a strong resilience to ethnic discrimination in participants with a strong Sami identity regardless of sex. In that study, the main outcome measures were mental health and well-being, and individual (personal) strength and cohesion (family) were protective factors [52]. These cultural factors may also be protective with regard to pain experiences in a similar population. Further, as previously mentioned, a tendency to not discuss illness among Sami people has been reported [16]. Consequently, cultural differences could have affected how respondents reported pain in the present study, and pain may have been underreported by Sami responders. Studies of cultural differences in illness perception and pain attitudes are only in their preliminary stages and limited research has been reported [53]. However, with such small differences between ethnic groups in reported pain, as shown in our study, the question remains whether research on pain in ethnic groups is justified.

Another possible reason for the minor difference in reported pain is the improvement of health and living conditions in the Sami population. In general, socioeconomic conditions and education have improved in the northern regions, and findings of significant health differences between the Sami and the majority populations are scarce [24]. In accordance with some of our results, Eriksen et al. [22] found no ethnic difference in general persistent pain and number of pain sites; however, the respondents in that study were 18–69 years old. Our results are also in line with studies on Sami and non-Sami adolescents [23]. This study adds to the literature by demonstrating minor ethnic differences in reports of persistent pain in an elderly population.

The strength of this study is that it is part of a national epidemiological programme, aimed at investigating health and living conditions in the Sami population. The Sami parliament in Norway and the municipalities have taken active part in the planning and formation of the SAMINOR study [25]. The study comprises a large number of participants from ten different municipalities with ethnically mixed populations, and up to 38.2% of the respondents self-identified as Sami [25]. The participation rate in our study was 44.5%, which is comparable to other Norwegian studies [2,26]. However, there are also some limitations. The study sample is comprised of more male than female respondents, and the respondents were younger and had higher income and education levels compared with the non-respondents, which may have caused selection bias. The prevalence of persistent pain reported in this study may, therefore, not be representative of the total population. As few participants responded to the questions about pain intensity and location, results from these analyses may be subject to selection bias; for example, they could reflect the experience of respondents with the most pain. The NRS used to measure pain severity has been found easy to use, especially in elderly populations [27]. However, determining the “mean” pain for the previous week may be challenging and could reflect more than just the magnitude of pain, for example fear related to pain [54]. Our question about pain location gave only one possible answer for the responders, and consequently may have provided incorrect results with respect to respondents with multiple pain sites. Further, the use of a dichotomised variable with a yes/no alternative for persistent pain may have excluded participants with fluctuating pain. However, associations between persistent pain and covariates were as expected. We saw trends in the relationship between household income and persistent pain, and a higher number of respondents could have revealed an interaction. A further study limitation involved the use of self-report data concerning persistent pain and mental health, as these data reflect an individual’s subjective interpretations; however, such information is only available as self-report data. The identification of ethnicity using self-reports may be questionable as, for example, respondents with a Sami background could have identified otherwise. Ethnicity criteria have been subject to investigation in a study using SAMINOR survey data [14], in which the authors indicated that self-identification was ethically preferable, as has also been recommended by the United Nations [14]. Finally, as this is a cross-sectional study, causal relationships cannot be determined.

### Conclusion

This study revealed only minor differences in persistent pain between Sami and non-Sami responders. The only significant difference was that elderly Sami women reported less pain than non-Sami women did. Socioeconomic factors, marital status, and mental health did not influence the relationship between ethnicity and health. Similar living conditions and cultural features may explain this, and should be further investigated. The findings suggest that awareness of the importance of socioeconomic conditions, education, and sex is central to understanding persistent pain in areas with a multicultural population. Health care providers should recognise that socioeconomic factors, sex, and ethnic background may interact in the development of persistent pain.
